# Evaluation of the prevalence of cardiometabolic disorders (diabetes, hypertension, and hyperlipidemia) diagnosed, undiagnosed, treated, and treatment goal in the elderly: Bushehr Elderly Health Program (BEH)

**DOI:** 10.1186/s12902-024-01561-0

**Published:** 2024-03-05

**Authors:** Mahbube Ebrahimpur, Erfan Mohammadi-Vajari, Yasaman Sharifi, Leila Ghotbi, Masoumeh Sarvari, Aryan Ayati, Baran Hashemi, Zhaleh Shadman, Pouria Khashayar, Afshin Ostovar, Noushin Fahimfar, Gita Shafiee, Elnaz Shahmohamadi, Tahereh Yavari, Iraj Nabipour, Bagher Larijani, Moloud Payab, Farshad Sharifi

**Affiliations:** 1 Endocrinology and Metabolism Research Center, Endocrinology and Metabolism Clinical Sciences Institute, Tehran University of Medical Sciences, Tehran, Iran; 2 Elderly Health Research Center, Endocrinology and Metabolism Population Sciences Institute, Tehran University of Medical Sciences, Tehran, Iran; 3 School of Medicine, Guilan University of Medical Sciences, Rasht, Iran; 4 Department of Radiology, School of Medicine, Iran University of Medical Sciences, Tehran, Iran; 5 Department of Internal Medicine, Shariati Hospital, Tehran University of Medical Sciences, Tehran, Iran; 6 Non-Communicable Diseases Research Center, Endocrinology and Metabolism Population Sciences Institute, Tehran University of Medical Sciences, Tehran, Iran; 7 Rajaie Cardiovascular Medical and Research Center, Iran University of Medical Science, Tehran, Iran; 8 Institute of Cardiovascular and Medical Sciences, University of Glasgow, Glasgow, UK; 9 Osteoporosis Research Center, Endocrinology and Metabolism Clinical Sciences Institute, Tehran University of Medical Sciences, Tehran, Iran; 10 Department of Epidemiology and Biostatistics, School of Public Health, Tehran University of Medical Sciences, Tehran, Iran; 11 Chronic Diseases Research Center, Endocrinology and Metabolism Population Sciences Institute, Tehran University of Medical Sciences, Tehran, Iran; 12 The Persian Gulf Marine Biotechnology Research Center, The Persian Gulf Biomedical Sciences Research Institute, Bushehr University of Medical Sciences, 7514633196 Bushehr, Iran

**Keywords:** Diabetes, Hypertension, Hyperlipidemia, Elderly

## Abstract

As the population ages, the global burden of cardiometabolic disorders will increase. This study aimed to investigate the prevalence of cardiometabolic disorders (diabetes, hypertension, and hyperlipidemia) in elderly and to evaluate the effects of various variables including age, sex, education, marital status, smoking, income, physical activity, dementia and depressed mood on untreated cardiometabolic disorders. This was a cross sectional study conducted in Bushehr Elderly Health Program. A total 2381 participants were included. Medical data were collected by trained interviewers. The mean age of the study participants was 69.34 years. Proportions of diabetes, hypertension, hyperlipidemia and hypercholesterolemia were 43.25%, 75.71%, 64.74% and 35.31% respectively. Untreated diabetes prevalence was higher for males (OR = 1.60, 95%CI = 1.20–2.15), older adults (OR = 1.02, 95%CI = 1.00–1.05), and pre-frail status (OR = 0.69, 95%CI = 0.52–0.92). Males (OR = 2.16, 95%CI = 1.64–2.84) and current smokers (OR = 1.42, 95%CI = 1.05–1.93), in contrast to married participants (OR = 0.25, 95%CI = 0.08–0.78), people with higher education levels (OR = 0.51, 95%CI = 0.29–0.89) and dementia (OR = 0.78, 95%CI = 0.61–1.00) were more likely to have untreated HTN. Untreated dyslipidemia is more common in smokers (OR = 1.78, 95%CI = 1.19–2.66) and males (OR = 1.66, 95%CI = 1.21–2.27), while untreated hypercholesteremia is more common in males (OR = 3.20, 95%CI = 1.53–6.69) and is reported lower in people with dementia (OR = 0.53, 95%CI = 0.28–1.01).

## Introduction

Human life expectancy continues to increase rapidly. In fact, life expectancy has increased at least by 20 years since 1950 worldwide. Nowadays, countries all around the world is facing increase in elderly and their complications. It is anticipated that in 2030, 1 in 6 people in the world will be aged over 60 years [1].

Chronic disorders affecting the elderly such as diabetes, hypertension, and hyperlipidemia have a considerable impact on health care costs. Presently, with improvement of global basic health measures, cardiovascular disorders are the most common cause of death. But hopefully, many of these cardio metabolic disorders are preventable or manageable. Increasing cardio metabolic diseases in elderlies could be reasoned with numbers of etiologies. In summary, the two major pathophysiological cause of age-related disease are chronic, low-grade inflammation and increased cellular oxidative stress [2–4].

With increased longevity, the prevalence of many chronic diseases rises up. Canadian Community Health Survey (CCHS) state that about 37% of elderly report having at least two of the ten common chronic diseases [5].

Diabetes mellitus (DM) is the most common metabolic disorder that based on the international diabetes federation state, globally, 1 in 11 adults has diabetes. 90% of diabetic patients have type 2 DM which is commonly diagnosed later in life [6]. The Middle East and North African regions have the world’s second highest age-adjusted diabetes prevalence, with nearly 49% still undiagnosed [7]. Thus, effective prevention, early diagnosis and treatment could be cost-effective for countries.

Hypertension (HTN) mentioned as the most common preventable and potentially reversible risk factor of cardiovascular disease [8]. Hypertension affects approximately 20% of the global population, and the prevalence of HTN is currently between 14.7 and 26.4% in various Eastern Mediterranean countries, based on the WHO report [9, 10]. In America, only 37% of adults with hypertension have their condition properly under control [11]. One of the most important risk factors for developing HTN is increasing age. Aging is inevitable but early diagnosis, life style change, appropriate pharmacological treatment which targeting the mechanisms of HTN could diminish adverse effects.

Dyslipidemia is attributed to more than half of all cases of coronary heart disease worldwide. According to World Health Organization (WHO) estimates, the worldwide prevalence of increased total cholesterol in adults (≥ 5.0 mmol/l) was estimated to be 39% [12]. Prevalence of hyperlipidemia, another important cardio metabolic disorders increase with age. Because of its causative role in the development of atherosclerosis and often clinically asymptomatic before subsequent cardiovascular disease, screening and treatment of hyperlipidemia is important [13]. Primary prevention in elderly is important because most of first cardiovascular events occur after 65 years old [14]. Management of hyperlipidemia needs accurate risk stratification of elderlies. The value of secondary prevention clarified by studies that have shown the benefits of lipid-lowering drugs on mortality of patients [15]. Despite the high importance of screening for metabolic risk factors such as diabetes, blood pressure and dyslipidemia, many of these disorders remain undiagnosed. For this reason, for example, in the case of diabetes, a screening recommendation has been changed to the age of over 35. By early diagnosis of these risk factors and their treatment, the possibility of their complications such as stroke, cardiovascular events or chronic complications of diabetes will be reduced or delayed.

## Materials and methods

### Sampling and setting

This is a cross-sectional approach to data gathered from the second phase of Bushehr Elderly Health Program (BEHP). The BEHP was community-based prospective in Bushehr, a provincial capital city in the south of Iran with 3000 participants. The sampling of this study was a multistage stratified random sampling method accomplished in neighborhoods of Bushehr. The second phase of this study (considered musculoskeletal and cognitive outcomes) was started in 2015. In this phase, more than 2700 participants of phase I was enrolled again. The study design and protocol were explained separately [16].

### Data gathering

The participants were interviewed by trained interviewers to collect data on their socio-demographic status, lifestyle (physical activity and smoking), medical history, and medication use. The questionnaire used for data gathering has been previously published elsewhere [16]. Anthropometric measurements were performed manual based on the National Health and Nutrition Examination Survey (NHANES). Assessments of anthropometric parameters, physical activity level (PAL), and blood pressure were discussed in detail separately. By using two tools (Mini-Cog and categorical verbal fluency test; CFT) dementia was evaluated and depression mood assessed by Patient Health Questionnaire (PHQ-9) [16–18]..

### Biochemical measurements

To determine the values of fasting blood sugar (FBS), hemoglobin A1c (HbA1c), and lipid profile, blood sample was taken after a 12 h overnight fast. FBS was measured by the enzymes (glucose oxidase) colorimetric method using a commercial kit (Pars Azmun, Karaj, Iran). HbA1c was also measured by Boranate affinity method using a CERA-STAT system (CERAGEM MEDISYS, chungcheongnam-do, Korea). Lipid profile and total cholesterol were measured by enzymatic (CHOD-PAP) colorimetric method using a commercial kit (Pars Azmun).

Diabetes was defined on the basis of at least one of the following: A1C ≥ 6.4% or fasting plasma glucose ≥ 126 mg/dl or previous diagnosed diabetes history or diabetes medication use [19]. Hyperlipidemia was defined as the presence of one of the following: total cholesterol ≥ 200 mg/dl or LDL-C ≥ 130 mg/dl or triglyceride ≥ 150 mg/dl or hyperlipidemia medication use. Hypercholesterolemia was defined as LDL-C ≥ 130 mg/dl or hypercholesterolemia medication use [20].

### Statistical analysis

Concerning statistical analysis, results were presented as mean ± standard deviation (SD) for quantitative variables and were summarized by frequency (percentage) for categorical variables. Categorical variables were compared using the chi-square test. The logistic regression models were used to evaluate the association between various variables with untreated hypertension, diabetes, dyslipidemia and hypercholesterolemia. The proportions were carried out age-standardization based on World Health Organization (WHO) population 2000–2025. For the statistical analysis, the software STATA version 12 for windows (StataCorp, Texas 77,845 USA) was used. *P* values < 0.05 was considered as statistically significant.

## Results

Out of 2772 total planned study participants 2381 participated in the study making the overall response rate of the study 85.9%. In this study, all of the respondents were living in urban area.

Among the total study participants about 1138 (47.8%) were male and 1243 (52.2%) were female, which makes sex ratio 0.91. The mean age of the study participants was 69.34 years ± 6.40 SD and about 589 (24.7%), 934 (39.2%), 371 (15.6%), 275 (11.5%), 143 (6.0%) and 69 (2.9%) of the study participants fell in the age groups between 60 and 64, 65–69, 70–74, 75–79, 80–84 and ≥ 85 years, respectively. 188 (8.01%) and 1013 (43.14%) participants were in frail and pre-frail status, respectively. Out of the total study participants, 1829 (76.8%) were currently married, 784 (32.9%) were illiterate, and 1641 (68.9%) had body mass index of greater or equal to 25. In addition, 1047 (44.14%), 829 934.85%), and 496 (20.91%) participants were non-smokers, past smokers, and current smokers, respectively. 2348 (98.7%) participants did not drink and 31(1.30%) participants were drinkers. 1109 (46.6%) had cognition impairment and, 1893 (79.5%) had low physical activity (Table 1).

**Table 1:** Socio-demographic and laboratory characteristics of the study participants

	Total participants
**Sex, (2381)**	
Female	1243 (52.2)
Male	1138 (47.80)
Age (y/o)	69.34 ± 6.40
**Age, (2381)**	
60–64	589 (24.74)
65–69	934 (39.23)
70–74	371 (15.58)
75–79	275 (11.55)
80–84	143 (6.01)
≥85	69 (2.90)
**Frailty**	
Non-frail	1147 (48.85)
Pre-frail	1013 (43.14)
Frail	188 (8.01)
Waist circumference (cm)	98.86 ± 11.74
BMI (kg/m^2^)	27.53 ± 4.89
**BMI (2379)**	
<18.5	45 (1.89)
18.5–24.9	693 (29.13)
25–29.9	1009 (42.41)
≥30	632 (26.57)
**Education, (2379)**	
Illiterate	784 (32.96)
Primary School	873 (36.70)
High School	215 (9.04)
Diploma	320 (13.45)
Academic	187 (7.86)
**Marital status (2381)**	
Single	19 (0.82)
Married	1829 (76.82)
Divorced	19 (0.80)
Widow	514 (21.58)
**Smoking (2374)**	
Non-smoker	1047 (44.14)
Past smoker	829 (34.95)
Current smoker	496 (20.91)
**Drinking (2379)**	
No	2348 (98.70)
Yes	31 (1.30)
Cognition impairment (2379)	1109 (46.62)
**Physical activity (2381)**	
Low	1893 (79.50)
Moderate	488 (20.50)
FBS (mg/dl)	106.12 ± 42.56
Cholesterol (mg/dl)	182.21 ± 44.24
HDL-C (mg/dl)	45.97 ± 11.23
LDL-C (mg/dl)	109.44 ± 109.44
Triglyceride (mg/dl)	135.76 ± 70.15
**Blood pressure (mmHg)**	
Systolic	139.74 ± 19.30
Diastolic	81.57 ± 8.64

*Abbreviations* BMI, Body Mass Index; FBS, Fasting Blood Sugar; HDL-C, high-density lipoprotein-cholesterol; LDL-C, low-density lipoprotein-cholesterol

The present study found that about 1,692 (71.1%) of patients had a history of hypertension or hypertensive, 1,135 (47.7%) had a history of diabetes mellitus or high serum glucose, 1,721 (72.3%) had a history of hyperlipidemia or high serum lipids and 1,507 (63.3%) had a history of hypercholesterolemia or high serum cholesterol (Table 2). Among diabetic patients, 460 (56.51%) were treated with metformin. The number of patients treated with other antidiabetic medications is mentioned as follows: 357 (43.56%) patients with Glibenclamide, 17 (2.09) with Repaglinide, 49 (6.02) with pioglitazone, 28 (3.44%) with Gliclazide, and 12 (1.47) with sitagliptin. Moreover, none of the participants used SGL2 inhibitors. Prevalence of standardized proportions of diabetes, hypertension and dyslipidemia of study participants were presented in Table 3.

**Table 2:** Crude prevalence of diabetes, hypertension and dyslipidemia of study participants

	Diabetes (*n* = 2379)	Hypertension (*n* = 2381)	Hyperlipidemia (*n* = 2381)	Hypercholesterolemia (*n* = 2381)
Male	516 (45.34)	790 (69.42)	731 (64.24)	606 (53.25)
Female	619 (49.88)	902 (72.57)	990 (79.65)	901 (72.49)
*P*	0.027	0.091	< 0.001	< 0.001
Total	1,135 (47.71)	1,692 (71.06)	1,721 (72.28)	1,507 (63.29)
60–69	754 (49.54)	1,044 (68.55)	1,139 (74.79)	986 (64.74)
70–79	289 (44.81)	485 (75.08)	447 (69.2)	403 (62.38)
≥80	92 (43.4)	163 (76.89)	135 (63.68)	118 (55.66)
*P*	0.055	0.001	< 0.001	0.031

**Table 3:** Age-standardized proportions based on WHO population of diabetes, hypertension and dyslipidemia of study participants

	Diabetes (*n* = 2379)	Hypertension (*n* = 2379)	Hyperlipidemia (*n* = 2379)	Hypercholesterolemia (*n* = 2379)
Female	44.33 (38.11–50.73)	76.62 (70.77–81.60)	71.18 (64.66–76.92)	44.26 (37.91–50.80)
60–69	51.07 (47.63–54.50)	69.70 (66.44–72.77)	82.80 (80.04–85.24)	41.56 (38.21–44.99)
70–79	46.17 (40.40–52.04)	77.85 (72.62–82.32)	76.09 (70.60–80.83)	41.28 (35.61–47.19)
≥80	42.03 (32.39–52.33)	77.68 (67.90–85.14)	66.51 (56.05–75.55)	46.11 (36.08–56.46)
Male	42.50 (35.53–49.79)	74.95 (68.51–80.46)	58.28 (50.91–65.31)	24.79 (19.52–30.93)
60–69	47.59 (43.88–51.33)	66.54 (62.93–69.97)	66.98 (63.40–70.37)	26.89 (23.70–30.33)
70–79	41.16 (35.79–46.76)	73.65 (68.45–78.27)	63.78 (58.25–68.96)	32.48 (27.44–37.97)
≥80	41.91 (31.00–53.67)	77.40 (66.44–85.56)	54.03 (42.36–65.28)	21.11 (13.43–31.60)
Total	43.25 (38.59–48.04)	75.71 (71.47–79.50)	64.74 (59.88–69.32)	35.31 (30.85–40.04)
60–69	49.48 (46.95–52.01)	68.32 (65.91–70.62)	75.64 (73.41–77.74)	34.87 (32.50–37.32)
70–79	43.52 (39.55–47.57)	75.63 (72.00–78.93)	69.58 (65.69–73.20)	36.62 (32.79–40.63)
≥ 80	41.73 (34.40–49.44)	77.42 (70.38–83.19)	60.32 (52.53–67.62)	34.86 (27.90–42.53)

The present study found that the overall magnitude of undiagnosed diabetes mellitus in the study area was 25.5% (28.9% in male vs. 22.6% in female, *P* = 0.016). There was no statistically significant difference in terms of undiagnosed diabetes by age groups. The prevalence of untreated diabetes mellitus was 50.4% (55.6% in male vs. 46.0% in female, *P* = 0.001). The prevalence of untreated diabetes mellitus was relatively high with the increasing of age of participants and the highest prevalence was observed within the group of study participants aged ≥ 80 years (*P* = 0.041).

This study showed that the prevalence of untreated hypertension was 25.1% (32.1% in male vs. 18.9% in female, *P* < 0.001) with the highest prevalence within the group of ≥ 80 years (*P* = 0.019).

The prevalence of untreated dyslipidemia and untreated hypercholesterolemia were 55.1% and 49.3%. There was no statistically significant difference in terms of untreated dyslipidemia and untreated hypercholesterolemia by age and sex (Tables 4 and 5).

**Table 4:** Crude prevalence of undiagnosed- untreated diabetes mellitus, hypertension, dyslipidemia and hypercholesterolemia

	Undiagnosed diabetes (*n* = 1135)	Untreated diabetes (*n* = 1135)	Untreated hypertension (*n* = 1692)	Untreated dyslipidemia (*n* = 1721)	Untreated hypercholesterolemia^1^ (*n* = 1507)	Untreated hypercholesterolemia^2^ (*n* = 242)
Male	149 (28.88)	287 (55.62)	254 (32.15)	420 (57.46)	299 (49.34)	79 (94.05)
Female	140 (22.62)	285 (46.04)	171 (18.96)	529 (53.43)	444 (49.28)	140 (88.61)
*P*	0.016	0.001	< 0.001	0.097	0.981	0.170
Total	289 (25.46)	572 (50.4)	425 (25.12)	949 (55.14)	743 (49.3)	219 (90.5)
60–69	182 (24.14)	365 (48.41)	267 (25.57)	626 (54.96)	478 (48.48)	148 (90.8)
70–79	78 (26.99)	150 (51.9)	105 (21.65)	244 (54.59)	203 (50.37)	54 (90.0)
≥ 80	29 (31.52)	57 (61.96)	53 (32.52)	79 (58.52)	62 (52.54)	17 (89.47)
*P*	0.243	0.041	0.019	0.707	0.623	0.972

*Abbreviation* LDL-C, low-density lipoprotein-cholesterol

^1^Analyzed by cut-off LDL cholesterol level ≥ 130 mg/dl

^2^Analyzed by cut-off LDL cholesterol level ≥ 160 mg/dl

**Table 5:** Age-standardized proportions based on WHO population of undiagnosed- untreated diabetes mellitus, hypertension, dyslipidemia and hypercholesterolemia

	Undiagnosed diabetes	Untreated diabetes	Untreated hypertension	Untreated dyslipidemia	Untreated hypercholesterolemia^1^	Untreated hypercholesterolemia^2^
Female	29.62 (20.79–40.31)	62.68 (54.54–7015)	20.16 (14.65–27.09)	58.64 (50.74–66.12)	55.42 (47.31–63.26)	89.45 (78.01–95.30)
60–69	22.84 (19.03–27.15)	43.64 (38.93–48.47)	19.36 (16.32–22.81)	53.26 (49.45–57.03)	49.03 (45.05–53.02)	89.12 (81.61–93.79)
70–79	23.95 (16.95–32.70)	53.11 (44.29–61.74)	15.75 (11.55–21.11)	52.81 (45.94–59.57)	49.23 (42.14–56.35)	88.98 (69.61–96.60)
≥80	33.52 (19.87–50.62)	70.97 (57.32–81.65)	22.17 (13.77–33.70)	62.28 (49.37–73.65)	59.45 (46.26–71.40)	89.72 (69.42–97.10)
Male	35.19 (25.09–46.83)	63.71 (53.20–73.05)	37.26 (29.64–45.57)	55.70 (45.69–65.27)	47.87 (37.54–58.37)	97.02 (91.50–99.00)
60–69	25.76 (21.34–30.75)	54.28 (48.89–59.57)	33.18 (29.02–37.63)	57.21 (52.64–61.66)	47.63 (42.59–52.72)	94.00 (82.90–98.06)
70–79	35.08 (27.17–43.91)	57.15 (48.42–65.45)	26.00 (20.72–32.08)	58.38 (51.36–65.08)	53.41 (45.92–60.75)	91.52 (69.36–98.09)
≥80	37.38 (21.87–56.01)	68.57 (50.87–82.13)	42.86 (30.67–55.98)	54.24 (38.36–69.31)	45.62 (29.68–62.51)	100
Total	32.31 (25.43–40.05)	63.28 (56.79–69.32)	28.41 (23.62–33.74)	57.79 (51.47–63.87)	52.82 (46.19–59.35)	92.34 (84.80–96.30)
60–69	24.05 (21.11–27.25)	48.18 (44.60–51.78)	25.66 (23.07–28.44)	54.97 (52.05–57.85)	48.55 (45.42–51.68)	90.74 (85.12–94.38)
70–79	29.44 (23.98–35.55)	55.06 (48.87–61.11)	21.01 (17.49–25.01)	55.53 (50.62–60.34)	51.23 (46.06–56.37)	90.00 (77.34–95.95)
≥80	35.37 (24.61–47.85)	70.12 (59.31–79.07)	32.11 (24.55–40.75)	59.37 (49.10–68.88)	54.45 (43.78–64.73)	93.67 (79.00–98.31)

*Abbreviation* LDL-C, low-density lipoprotein-cholesterol

^1^Analyzed by cut-off LDL cholesterol level ≥ 130 mg/dl

^2^Analyzed by cut-off LDL cholesterol level ≥ 160 mg/dl

### Factors associated with untreated diabetes mellitus, hypertension, dyslipidemia and hypercholesterolemia

Multivariate logistic regression analysis of factors associated with untreated diabetes mellitus found that the age of the participants, being male and pre-frail status were the main factors that revealed statistically significant association with untreated diabetes mellitus. For every decade of age over 60, the odds ratio of untreated DM increases by 1.02 times (95%CI = 1.00–1.05). The occurrence of untreated DM among male study participants were about 60% more likely (OR = 1.60, 95%CI = 1.20–2.15) compared to female participants (Table 6).

**Table 6:** Association of characteristic of the participants with untreated diabetes

	OR	95%CI	P-value
Age	1.02	(1.00–1.05)	0.041
**Frailty**			
Pre-frail	0.69	(0.52–0.92)	0.01
Frail	0.70	(0.37–1.32)	0.28
Sex (reference group: female)	1.60	(1.20–2.15)	0.002
**Marital status (reference group: single)**			
Married	0.42	(0.08–2.37)	0.327
Divorced	0.80	(0.08–7.51)	0.844
Widow	0.54	(0.10–3.07)	0.489
**Education (reference group: Illiterate)**			
Primary School	1.15	(0.85–1.56)	0.377
High School	1.02	(0.63–1.64)	0.940
Diploma	1.05	(0.66–1.66)	0.845
Academic	1.63	(0.91–2.90)	0.097
**Smoking**			
Past smoker	1.06	(0.81–1.41)	0.63
Current smoker	1.17	(0.83–1.64)	0.34
Drinking	1.75	(0.51–5.95)	0.36
**Income (reference group: <155$)**			
155–312$	0.78	(0.57–1.07)	0.130
≥ 312$	1.01	(0.67–1.52)	0.960
Low Physical Activity	1.33	(0.96–1.86)	0.090
Dementia	1.00	(0.76–1.30)	0.985
Depressed mood	1.49	(1.00–2.23)	0.053

*Abbreviations: OR*, odds ratio; 95%CI, 95% confidence level

Also, the current study found that sex, marital status, educational status, current smoking and, dementia revealed statistically significant association with untreated hypertension (Table 7). The occurrence of untreated hypertension was higher in men (OR = 2.16, 95%CI = 1.64–2.84) and current smokers (OR = 1.42, 95%CI = 1.05–1.93), was lower in married and widow participants (OR = 0.25, 95%CI = 0.08–0.78 and, OR = 0.22, 95%CI = 0.07–0.71 respectively), participants with higher educational level (OR = 0.51, 95%CI = 0.29–0.89) and with dementia (OR = 0.78, 95%CI = 0.61–1.00).

**Table 7:** Association of various variables with untreated hypertension

	OR	95%CI	P-value
Age	1.00	(0.98–1.02)	0.67
**Frailty**			
Pre-frail	0.78	(0.58–1.03)	0.08
Frail	0.67	(0.37–1.23)	0.20
**Marital status (reference group: single)**			
Married	0.25	(0.08–0.78)	0.02
Divorced	0.23	(0.04–1.25)	0.09
Widow	0.22	(0.07–0.71)	0.01
**Education (reference group: Illiterate)**			
Primary School	0.79	(0.59–1.05)	0.10
High School	0.84	(0.54–1.30)	0.43
Diploma	0.63	(0.41–0.98)	0.04
Academic	0.51	(0.29–0.89)	0.02
**Smoking**			
Past smoker	0.91	(0.70–1.18)	0.51
Current smoker	1.42	(1.05–1.93)	0.02
Drinking	1.10	(0.41–2.92)	0.84
**Income (reference group: <155$)**			
155–312$	0.84	(0.62–1.14)	0.27
≥ 312$	0.83	(0.57–1.23)	0.36
Low physical activity	1.18	(0.87–1.60)	0.28
Dementia	0.78	(0.61–1.00)	0.05
Depressed mood	0.92	(0.62–1.38)	0.70

*Abbreviations* OR, odds ratio; 95%CI, 95% confidence level

The study also found that the occurrence of untreated dyslipidemia was about 1.5 times higher among men (OR = 1.66, 95%CI = 1.21–2.27) and both former and current smokers (OR = 0.72, 95%CI = 0.57–0.90 and OR = 1.70, 95%CI = 1.28–2.25, respectively) (Table 8).

**Table 8:** Association of various variables with untreated dyslipidemia

	OR	95%CI	P-value
Age	1.01	(0.98–1.03)	0.531
**Frailty**			
Pre-frail	0.93	(0.74–1.18)	0.599
Frail	0.81	(0.48–1.38)	0.458
Sex (reference group: female)	1.66	(1.21–2.27)	0.002
**Marital status (reference group: single)**			
Married	1.31	(0.34–5.00)	0.690
Divorced	0.65	(0.12–3.65)	0.628
Widow	1.50	(0.39–5.82)	0.558
**Education (reference group: Illiterate)**			
Primary School	0.87	(0.63–1.20)	0.384
High School	1.21	(0.69–2.12)	0.509
Diploma	0.86	(0.52–1.41)	0.541
Academic	1.92	(0.90–4.10)	0.092
**Smoking**			
Past smoker	0.72	(0.57–0.90)	0.005
Current smoker	1.70	(1.28–2.25)	0.001
Drinking	1.37	(0.53–3.53)	0.50
**Income (reference group: <155$)**			
155–312$	0.78	(0.55–1.10)	0.158
≥ 312$	0.93	(0.58–1.48)	0.748
Low physical activity	1.25	(0.84–1.86)	0.278
Dementia	1.07	(0.80–1.43)	0.666
Depressed mood	1.00	(0.64–1.57)	0.998

*Abbreviations* OR, odds ratio; 95%CI, 95% confidence level

Lastly, the current study found that the occurrence of untreated hypercholesterolemia was about 3 times higher among men (OR = 3.20, 95%CI = 1.53–6.69), 50% lower in participants with dementia (OR = 0.53, 95%CI = 0.28–1.01) and smokers (former smokers; OR = 0.006, 95%CI = 0.56–0.91/ current smokers; OR = 0.001, 95%CI = 1.20–2.18) (Table 9).

**Table 9:** Association of various variables with untreated hypercholesterolemia

	OR	95%CI	P-value
Age	0.97	(0.93–1.02)	0.293
**Frailty**			
Pre-frail	0.93	(0.73–1.20)	0.62
Frail	0.84	(0.48–1.46)	0.55
Sex (reference group: female)	3.20	(1.53–6.69)	0.002
**Marital status (reference group: single)**			
Married	1.91	(0.20–18.15)	0.573
Divorced	0.52	(0.03–8.73)	0.652
Widow	1.82	(0.19–17.84)	0.605
**Education (reference group: Illiterate)**			
Primary School	0.60	(0.31–1.14)	0.117
High School	0.87	(0.27–2.85)	0.82
Diploma	0.39	(0.14–1.07)	0.067
Academic	1	(0.56–1.53)	0.76
**Smoking**			
Past smoker	0.71	(0.56–0.91)	0.006
Current smoker	1.62	(1.20–2.18)	0.001
Drinking	1.88	(0.67–5.21)	0.22
**Income (reference group: <155$)**			
155–312$	0.91	(0.47–1.78)	0.786
≥ 312$	0.72	(0.30–1.75)	0.470
Low Physical Activity	0.82	(0.41–1.64)	0.577
Dementia	0.53	(0.28–1.01)	0.052
Depressed mood	0.83	(0.39–1.75)	0.621

*Abbreviations* OR, odds ratio; 95%CI, 95% confidence level

## Discussion

The present study assessed prevalence of diagnosed, undiagnosed and untreated cardio metabolic risk factors including diabetes, hypertension and hyperlipidemia.

Standardized proportions of diabetes, hypertension and hyperlipidemia and hypercholesterolemia of this study participants were reported to be 43.25% (38.59–48.04), 75.71% (71.47–79.50), 64.74% (59.88–69.32), and 35.31% (30.85–40.04) respectively.

Regardless of economic development, epidemiological, or demographic variability, all countries face an increasing burden of non-communicable diseases, including diabetes mellitus [21].

Esteghamati et al. stated that 7.7% of Iranian adults aged 25–64 years have diabetes, with half remaining undiagnosed. A further 16.8% of Iranian adults, or 4.4 million, have impaired fasting glucose [22]. In our study, 1,135 participants (47.7%) had a history of diabetes. This higher prevalence could be attributed to different study populations and fewer study subjects, as well as rising diabetes incidence since 2008.

Per Busaidi et al. [7], nearly 25.8 million people in the Middle East have diabetes, and many countries in this region have a higher prevalence of diabetes than the global average (8.8%) [23].

According to Animaw et al., urban dwellers, centrally obese, overweight, and hypertensive individuals have a higher risk of developing diabetes mellitus [21]. In our study, males, older people and pre-frail status had a higher prevalence of diabetes, contradicting the Animaw study. Frailty is a complex condition that cause physical and cognitive impairment in elderly. Mone et al. showed a significant correlation between frailty with diabetes and hypertension in older patients [24]. Otherwise, our study showed association between pre-frail status and diabetics, not hypertensive patients. For management of frailty and cognitive impairment, extended-release metformin has been introduced as a treatment to reduce cognitive impairment in frail women with hypertension and diabetes [25].

Chronic kidney disease (CKD) is a common disease in elderly with diabetes which associated with increased mortality and morbidity rate. Aging is an important predictor for development of CKD which 11% of individuals older than 65 years without underlining disease such as diabetes mellitus or hypertension suffering CKD [26]. Obesity through adipose-renal axis, is an independent risk factor for CKD [27]. Also aging by increasing adiponectin levels and insulin resistance affects the kidney function [28].

Several recent studies focused on the role of mineralocorticoid receptor (MR) in cardiovascular aging. The main role of MR is regulation of blood pressure by affection sodium retention in the kidneys [29]. Due to MR expression in extrarenal tissues, including heart, the hypothesis of a causal effect on cardiovascular aging has been proposed [30]. Hypertension is one of the leading causes of mortality and morbidity in the world [31]. The Middle East is an example of countries in the twenty-first century with a significant awareness, management, and control gap for high blood pressure [31].

Najafipour et al. reported that the prevalence of pre-HTN was 28.5%. The prevalence of HTN was 19.2% (13.9% diagnosed and 5.3% undiagnosed) in adult population in southeast Iran [10].

In our findings, the prevalence of untreated HTN was reported to be 28.41% (23.62–33.74), which is lower than the findings of Najafipour’s study of uncontrolled HTN in treated population (47.7%). This could be due to the different age populations that we assessed. As in the Najafipour study, the prevalence of HTN among people aged 75–80 years was reported to be higher than in younger populations ( 4% vs. 67.8%) [10].

According to Amini et al., those with a lower socioeconomic status who were more conscious of their blood pressure level had a higher prevalence of HTN. In spite of this, a high proportion of people with higher socioeconomic status are also treated and monitored for hypertension. This suggests that individuals with lower socioeconomic status are more likely to suffer an adverse hypertension event and receive less treatment and monitoring for it. This is because they have less access to resources [32]. Although this claim is not supported by our findings, and socioeconomic status did not affect hypertension prevalence or any other aforementioned cardio metabolic risk factors like diabetes or hypercholesteremia.

Following Kumar Das et al., older age, a non-vegetarian diet, and increasing BMI all have a significant influence on the prevalence of hypertension [33]. Owing to the fact that our findings confirmed a significant effect of smoking, dementia, and educational status on the prevalence of HTN, they did not support the age differences and didn’t evaluate diet and BMI in this community.

On the other hand, orthostatic hypertension (OHTN) role in cardiovascular disease is notable in elderly. Pasdar et al. showed systolic OHT was associated with increased risk of mortality, cognitive impairment, cardiovascular and brain comorbidities [34, 35].

Previous studies revealed that the prevalence of hypercholesterolemia in Iran is higher compared to other Asian countries [36, 37].Among Iranian people, meta-analysis by Tabatabai Malazy et al. found that the prevalence of hypercholesterolemia (≥ 200 mg/dl), hypertriglyceridemia (≥ 150 mg/dl), high levels of low-density lipoprotein cholesterol ([LDLC] [≥ 130 mg/dl]) and low levels of high density lipoprotein cholesterol ([HDLC] < 40 mg/dl in individuals aged ≥ 15 years, among males,<50 mg/dl in females), were 41.6% (36.1–47.0),46.0% (43.3–48.7), 35.5% (24.0–47.1), and 43.9% (33.4–54.4),among both sexes and in urban and rural areas, respectively [38]. The prevalence of untreated dyslipidemia and hypercholesterolemia among the elderly BEH population was reported to be 57.79% (51.47–63.87) and 52.82% (46.19–59.35), respectively, which is higher than the Tabatabai Malazy study. This could be due to two factors: first, the prevalence increased during the time of the study (this meta-analysis evaluated the data up to September 2011), and second, this study only included one ethnicity in Iran, whereas the previous study included all ethnicities in Iran in their meta-analysis study.

To the best of our knowledge, no earlier prospective cohort studies focused on assessment of the prevalence of cardiometabolic risk factors in the elderly population in Iran. There were also some limitations to this study. First, we used estimates based on the global population, primarily the US population. As such, they may be low or high compared to Iranian conditions. As a second limitation, the cohort study only included people over 60 years. As a result, it is difficult to compare the findings with those of other studies targeting the same cardio metabolic risk factors. Attrition bias caused by selection bias as well as recall problems of elderly people from the very first stage may be considered limitations of the current study. Another limitation of this study is that it only includes participants with adequate physical ability and excludes the elderly living in nursing homes, which has led to selection bias.

## Conclusions

Compared to other countries in this region, the prevalence of cardio metabolic diseases, such as diabetes, hypertension, and hyperlipidemia, was higher in our cohort population. Males and older adults were more likely to have untreated diabetes. Untreated HTN prevalence was higher for males and smokers, and lower for people with higher education levels and married participants. Untreated dyslipidemia is more common in smokers and males, while untreated hypercholesteremia is more common in males and is reported lower in people with dementia. These potential risk factors need to be evaluated further to confirm their impact on the prevalence of cardiometabolic diseases among the elderly. Additionally, future studies should examine screening plans for these cardiometabolic risk factors in younger adults as well as exploratory studies to determine the probable causes of patients who do not receive appropriate treatment despite confirmed diagnoses.

## Data Availability

All the data generated and/or analyzed during this study are included in this published article.
